# Nurse Farms, Hospitals, and Private Nursing Institutions

**Published:** 1887-03-12

**Authors:** 


### Nurse Farms, Hospitals, and Private Nursing Institutions.

The Views of Patients, Superintendents, and Private Nurses.

### By a Barrister and Private Patient.

Will you allow me, as an old subscriber to hospitals, to make a few observations on the subject-matter of Miss Manson's paper, reported in The Hospital? I am not a nurse farmer, but some years ago I was most carefully and efficiently nursed during a severe illness by nurses obtained from a private nursing institution, and several friends of mine have been similarly benefited; consequently I venture to differ from Miss Manson, and to say that, in my judgment, so far as the public are concerned, the system of letting out nurses on hire, which has been adopted by such institutions, is most beneficial, and the reverse of evil. It may be, that from some institutions inefficient nurses are occasionally sent out, but that does not prove that the system is bad, any more than the existence of some ignorant members of a profession proves the inutility of that profession as a whole. I entirely fail to see how such a nursing system can be unjust to the nurses attached to an institution. They are provided with regular employment, which they could not otherwise obtain for themselves, and if they were, as is suggested, unfairly treated by the proprietors of the institutions, they would not, I presume, leave the hospitals as they do, and seek employment at a nurse farm.

I am entirely opposed to our hospitals letting out nurses on hire; in my opinion, it is not right that the moneys of a hospital, which have been given to it by charitable persons to be applied for charitable purposes, should be applied towards carrying on a commercial speculation such as a nurse farm. We subscribers would never know how much of our money was diverted from the charitable work of the hospital to the keeping up of the farm. Neither is it right that the officials of a hospital, who are paid out of the charity moneys to do charitable work, should devote their time to the management of the farm. Neither man nor woman, however eminent, can serve God and Mammon. At present we know that the sick poor are nursed by as good nurses as the hospital managers can obtain, but if there be a farm attached to the hospital, then, in order to obtain customers, the best nurses will of course be let out on hire, and the inferior ones only will be set to work in the wards. If the farm nurses be, *in fact*, kept entirely distinct from the ward nurses, then the argument that the public will be best served by a nurse from a hospital farm on account of her being in touch with a hospital, will be unavailable.

If hospitals set up farms we shall have the edifying spectacle of our most noble institutions advertising for customers and struggling with one another, after the usual fashion of rival business establishments. Again, I suspect that unpleasant feelings will be developed amongst the nurses. At present, all work together in the wards on the same footing, but if there be a farm, each for herself will strive to enter its gate, as I can hardly imagine that a nurse would not prefer being sent out to work in a country house to remaining in the wards.

I should approve of the hospitals (if they could afford it) sending out nurses to tend gratuitously the sick poor in their homes, but in my opinion it is no more a function of a hospital to let out nurses on hire, than it would be to pay fixed salaries to its medical staff, and let them out in a similar way. If it were not possible for the rich to obtain nurses elsewhere, the case might be different. If it be argued that the hospitals are much in need of money, and that they are likely to make some by nurse-farming, I should answer, that agreeable result might possibly follow, but the hospital managers should bear in mind that it is also more than likely that some of their present subscribers will disapprove of this new departure from the path of charity, and will withdraw their support, so that the net result of the undertaking may be a loss. I must apologise, Sir, for the length of this letter, but I feel sure that the importance of the question and your sense of fairness will lead you to find room for it in The Hospital.

### By a Lady Superintendent of a Provincial Nursing Institution.

I have read the remarks from "Lady Superintendent" and "Trained Nurse" in your issue of this week, but, unfortunately, have not seen Miss Manson's, and, therefore, can only give my own experience and opinion independently, not pretending to answer anything she may have said. So far from making profits out of the nurses, at least in small institutions, it is very difficult to cover expenses, as there is always a portion of the year that they are not required; and as to the character and ability of the nurses, I can only say that I feel assured ours would compare favourably with any hospital nurses in the kingdom, or private nurses sent from hospitals. I have been connected with two hospitals, both of high standing for training, and many nurses sent out from both would not be accepted here. In one case, I knew of a wardmaid being put as night nurse for a few weeks, and then sent out as a private nurse. Large institutions may possibly reap profits by the habit of taking probationers, giving them six months' training (I am told, sometimes three months), and then sending them out; but the first fault lies with the hospitals taking nurses for so short a time. On my appointment, I found probationers training for six months, and at once had it extended to twelve; and as this is quite as expensive as taking trained nurses, and more uncertain as to their being good nurses, I prefer to engage them after being trained, some two years, some three; and there are always plenty of good nurses who, at least for a time, are glad to escape from the heavy work of hospital, and take their chance of good or bad in private work. Can it be wondered at? Although there is much to try the temper and constitution in private nursing, as there cannot be the same regularity as to hours off duty that there is in hospital work, but against that there is the lack of actual hard work, and the long strain daily of being on foot so many hours.

I know myself, while lady probationer, that from half-past seven a.m. to half-past twelve we never sat down. We then had half-an-hour for dinner, any ablutions or changing of aprons, etc. From one to three o'clock we were engaged round the wards with the visiting physicians or surgeons; afterwards, a round of different attentions to the patients, so that very seldom could those on duty sit down. Twice a week each nurse or lady probationer was allowed off for the afternoon, but other days the routine went on till nine p.m., then supper, then to prayers in chapel, when feeling thoroughly exhausted, producing, I believe, even in after-life, a carelessness in attending services.

As to the uniform, at an early stage of my experience I should have thrown in my lot against it; now I should vote in favour of it for many reasons. The nurses all tell me they get respect and attention wherever they go, that they would not get in their own dress; and I am sure it removes a temptation to over-dressing and over-spending; and could statistics be taken, it would be found that nurses with uniform have nearly all a savings-bank account, and those who wear their own dress have none. It certainly is an extra expense to the institutions, as I believe we have to pay as much as when no uniform is given, but I do think we get the benefit in the class of nurses we can command.

### By a Private Nurse.

Miss Manson's paper in your journal of February 19th, though admirable from a hospital matron's point of view, leads to such an unfair inference in regard to a large body of public servants, that you must allow us to enter our protest. I speak for private nurses generally, as well as for those with whom I am particularly connected—by far the largest body in London. The article we challenge says that "institutions which make any stipulation as to the length of time a nurse must have been trained are few and far between." As a fact, private institutions, whose actual existence depends so completely on public favour, are compelled to provide competent nurses, and the greatest number of their workers are those who have served for years in hospitals. Were statistics taken, we believe that the number of the better class of nurses who are induced to remain at their hospitals would be in the minority. They are not well paid, the work is hard, and the comforts few; though these have been better attended to of late years. That a woman should be sent by any institution for six months' or a year's training, is a very exceptional thing in London, and rarely happens elsewhere, unless she has shown capabilities for the work which make it certain it will be worth while her employers spending money on her training. If a great deal of tact and care is used in choosing nurses in a hospital, there is a great deal more exercised in choosing them for private work, where as individuals, often under prejudiced criticism, they have to satisfy the patients' friends and the doctor, as well as the fancies of an invalid, and the managers of the institution to whom they are reported at the termination of their engagement. Thus they are known and tested in a way impossible in a hospital, where, so long as the mass accomplishes the work, and conforms to the restrictions, the individual passes uncriticised.

We are apt to consider the recently-trained nurse as comparatively raw, for, owing to the number of patients in a hospital ward, she cannot be so well acquainted with all the details of each case as one who is engaged in private work. Provided neither age nor physical infirmity has incapacitated a nurse, or blunted her receptive faculties, we cannot understand how she becomes "rusty" when constantly employed. We do not find doctors get rusty in practice, and why should nurses do so? Any advance in science, any new remedy or invention, is at once discussed in our homes, or the medical man mentions it as a matter of professional interest to the nurse. Miss Manson says that "the women who are attracted to the work are of a class whose intelligence and education make them capable of being trained to a very high standard of proficiency." Surely this same intelligence will enable them to understand and carry out the new treatment, without having to resort to the hospital for three months to study it. That a cap and uniform may give a patient confidence in a nurse, I grant; but many who nurse patients accustomed to the sight of a lady's-maid without a cap, or those who object to being supposed to require a nurse, are glad to be able to dress to suit the patient, which they could not do if attached to a public hospital; and alas! the snowy cap and apron may cover a multitude of sins, even such as the absence of soap and water, and lack of repairs that are out of sight. In all communities there are some who do not live up to their outward appearance. No one hospital can boast of producing better or more useful nurses than are to be found engaged in private work. To the founder of the Association to which we belong, who in her nurses' estimation will always stand beside "Our Lady with the Lamp," belongs the honour of organising a system whereby properly qualified nurses may receive their hardly won earnings without being taxed to support the funds of either a hospital or a private institution, and this advantage leads many highly efficient nurses to join our ranks. To her nurses may with justice be applied the whole of Miss Manson's last paragraph. They are most carefully selected, supervised, and cared for; they constantly live, when off duty, among surroundings where nursing is highly considered, and hold an established position, and thus their self-respect and their consideration for the dignity of their profession are raised.

### MISS MANSON'S REPLY.

We think it well to state that Miss Manson's reply to her numerous critics will appear in The Hospital next week. We agree with her in thinking that the subject dealt with is so important to the public and the nursing profession generally, that it cannot be too freely discussed. We therefore propose to continue the discussion in these columns. Should there be other lady superintendents of hospitals or nursing institutions, sisters, nurses, and others, who desire to give their views and experience on the subject, we shall be glad to have their contributions with as little delay as possible.

